# Interactions between betaine, insulin resistance, and cognitive impairment in people with and without HIV

**DOI:** 10.3389/fimmu.2026.1878678

**Published:** 2026-07-15

**Authors:** Azin Tavasoli, Bin Tang, Mohammadsobhan S. Andalibi, Debra Cookson, Melanie Crescini, Margery A. Connelly, Scott L. Letendre, Ronald J. Ellis

**Affiliations:** 1 Department of Neurosciences, University of California, San Diego, San Diego, CA, United States; 2 Department of Psychiatry, University of California, San Diego, San Diego, CA, United States; 3 Department of Diagnostic R&D, LabCorp, Morrisville, NC, United States; 4 Department of Medicine, University of California, San Diego, San Diego, CA, United States

**Keywords:** cognition, diabetes mellitus, HIV, inflammation, insulin resistance

## Abstract

**Background:**

Metabolic dysfunction and neurocognitive decline share inflammatory pathways that may be amplified in people with HIV (PWH). Prior studies suggest insulin resistance correlates with cognitive decline, while gut-related metabolites, such as betaine, may be protective through metabolic regulation and anti-inflammatory effects. We investigated whether metabolic markers differentially associate with cognitive performance in PWH versus people without HIV (PWoH).

**Methods:**

We enrolled 200 participants, equally stratified by HIV and diabetes mellitus status, into four groups of 50. All completed neuropsychological testing yielded demographically corrected global T-scores (higher indicates better performance). Biomarkers included GlycA, Diabetes Risk Index (DRI), Lipoprotein Insulin Resistance Index (LP-IR), branched-chain amino acids, and betaine. Multiple linear regression examined associations between metabolic markers and T-scores, adjusting for covariates.

**Results:**

Participants had a mean age of 55.7 years (71.0% male). GlycA levels were higher in individuals with diabetes (p = 0.001). Higher DRI levels were associated with worse global T-scores (standardized β = -0.27, p = 0.009). Higher LP-IR was associated with worse cognitive performance in PWH, but not in PWoH (interaction p = 0.007). HIV moderated the association between betaine and global T-scores (interaction p = 0.01); higher betaine was associated with better cognitive performance (standardized β = 0.24, p = 0.02). Higher betaine correlated with lower LP-IR in PWH (standardized β = -0.47, p < 0.01) and lower DRI in all participants (ps < 0.05).

**Conclusion:**

Insulin resistance might play a prominent role in cognitive health in PWH than PWoH, suggesting HIV-related metabolic vulnerability is affecting brain health. Associations between betaine, which has immunomodulatory effects, and improved metabolic profiles suggest potential therapeutic targets to preserve cognitive function in PWH. These observational findings may reflect metabolic, dietary, or lifestyle-related factors and require confirmation in studies with detailed nutritional assessment.

## Introduction

Antiretroviral therapy (ART) has transformed HIV from a fatal illness into a chronic disease (1). As a result, people with HIV (PWH) are living longer and now face aging-related diseases that are common in the general population (2). Among these, metabolic dysfunction, including insulin resistance, obesity, and diabetes mellitus, is particularly prevalent and may disproportionately affect PWH due to persistent immune activation, ART-related toxicities, and sociodemographic factors (3). Both HIV infection and metabolic conditions have been linked to cognitive impairment, raising questions about their interaction and cumulative effects on brain health (4, 5). However, it remains unclear whether metabolic dysfunction is associated with cognitive performance in the same way in PWH and people without HIV (PWoH), or whether HIV-related immune and metabolic abnormalities confer additional vulnerability.

Insulin resistance (IR) is a metabolic condition in which cells become less responsive to insulin, leading to impaired glucose uptake and hyperinsulinemia (6). Over time, IR contributes to the development of type 2 diabetes mellitus, cardiovascular disease, and other metabolic disorders (7). It is also associated with chronic low-grade inflammation and may directly affect brain health through altered cerebral glucose metabolism, increased oxidative stress, and endothelial dysfunction (8). The Diabetes Risk Index (DRI) is a multi-analyte score that integrates lipoprotein particle size and number, as well as branched-chain amino acids (BCAAs), measured via nuclear magnetic resonance (NMR) spectroscopy, to estimate an individual’s long-term risk of developing type 2 diabetes. It reflects early pathophysiological changes before the onset of hyperglycemia and is sensitive to IR and beta-cell dysfunction (9). The lipoprotein IR Index (LP-IR) is another score that reflects IR based on lipoprotein particle concentrations and sizes. It combines six parameters from the lipoprotein profile to produce a continuous measure, with higher values indicating greater IR (10). LP-IR, ranging from 0 to 100, provides a quantitative estimate of IR and has strong correlations with the homeostatic model assessment of insulin resistance (HOMA-IR) (11). Since IR may impair cerebral glucose metabolism and neuronal health, LP-IR could serve as a sensitive marker linking metabolic dysfunction to neurocognitive impairment. While relationships between IR, inflammation, and neurological outcomes have been established in general populations, our study specifically investigated whether these associations are heightened in PWH compared to people without HIV, and how diabetes status modifies these relationships.

Systemic inflammation represents another potential pathway linking metabolic dysfunction and cognitive outcomes (12). GlycA is a composite inflammatory biomarker that reflects the concentration and glycosylation state of acute-phase proteins such as α1-acid glycoprotein, haptoglobin, α1-antitrypsin, and others (13, 14). Elevated GlycA levels have been associated with IR, diabetes risk, cardiovascular events, and all-cause mortality. In the context of HIV, GlycA may also reflect persistent immune activation and inflammation despite viral suppression (15, 16). Elevated GlycA levels were associated with greater odds of neurocognitive impairment in PWH (16).

Despite substantial evidence connecting metabolic health with cognitive decline in the general population, the biological mechanisms linking metabolic dysfunction and cognition in the context of HIV are poorly defined. In particular, whether established metabolic markers such as DRI, LP-IR, or GlycA operate through the same pathways in PWH as in PWoH is unknown, or whether HIV-related inflammation and diabetes confer unique vulnerabilities. Our study goes beyond prior work by directly testing whether the predictive value of these systemic metabolic markers differs by HIV and diabetes status.

The gut microbiome has emerged as a new and potentially modifiable mediator of metabolic disease (17). Betaine, trimethylamine N-oxide (TMAO), and choline influence host lipid metabolism, insulin sensitivity, and inflammation (17). Betaine is derived primarily from dietary intake and endogenous hepatic oxidation of choline (18). Although gut microbial pathways contribute to its catabolism to trimethylamine, betaine should not be considered a metabolite produced primarily by the gut microbiota. Betaine is intricately involved in lipid metabolism, as evidenced by its ability to attenuate hepatic triglyceride accumulation (19). Plasma betaine tends to correlate negatively with triglycerides and non-HDL cholesterol and positively with HDL cholesterol (17). Low circulating betaine appears to be associated with an elevated risk of developing type 2 diabetes mellitus (17, 20). Beyond metabolic regulation, betaine also contributes to brain health through its role as a methyl donor in one-carbon metabolism, supporting homocysteine clearance, reducing oxidative stress, and protecting neurons from excitotoxic and vascular injury (21). Betaine has anti-inflammatory effects by reducing pro-inflammatory cytokines such as TNF-α, IL-6, and IL-1β and by suppressing signaling pathways like NF-κB that drive chronic immune activation. It also helps reduce oxidative stress and supports mitochondrial and gut barrier function, which may contribute to improved metabolic regulation and lower systemic inflammation (22). While gut dysbiosis occurs in PWH, limited research has examined how microbial-associated metabolites relate to cognitive performance, especially in the presence of diabetes (23). Low betaine levels may predict cardiovascular disease (CVD) in people without diabetes and modify the extent to which TMAO confers an increased CVD risk (24).

BCAAs, such as valine, leucine, and isoleucine, are essential nutrients involved in energy metabolism and have also been implicated in IR and inflammation (25, 26). BCAAs interfere with insulin signaling by affecting the phosphorylation of key insulin pathway proteins, leading to reduced glucose uptake by muscle and liver cells (27). Elevated BCAA levels have been associated with obesity, type 2 diabetes, and CVD (28). Importantly, BCAAs may also impact brain health by altering neurotransmitter synthesis (e.g., competition with tryptophan for transport across the blood–brain barrier, reducing central serotonin availability) and by promoting neuroinflammatory cascades that contribute to cognitive decline. Yet, their role in the development of cognitive dysfunction, particularly in PWH and metabolic disease, remains underexplored (29).

Based on these interrelated pathways, we hypothesized that: 1) markers of metabolic dysfunction (DRI, LP-IR) would have stronger negative associations with cognitive performance in PWH compared to PWoH; 2) inflammation (GlycA) would be associated with metabolic dysfunction and cognitive impairment, particularly in those with comorbid HIV and diabetes; 3) betaine and BCAAs would have different associations with cognitive performance depending on HIV and diabetes status. This investigation aims to identify whether HIV infection and metabolic dysfunction synergistically affect brain health. Clarifying these relationships may enable clinicians to better stratify cognitive risk in PWH, informing targeted screening and proactive interventions to mitigate metabolic and cognitive deterioration. To better understand the specific metabolic and inflammatory mechanisms that may mediate cognitive impairment in PWH, we selected biomarkers that reflect distinct yet interconnected aspects of metabolic dysregulation, systemic inflammation, and gut microbiome involvement. Collectively, these biomarkers provide a multifaceted profile of metabolic, inflammatory, and microbiome-driven processes, enabling a more nuanced understanding of how metabolic dysfunction uniquely contributes to cognitive impairment in PWH compared to PWoH.

## Materials and methods

### Participants

The HIV Neurobehavioral Research Center (HNRC) leads a prospective, observational cohort of neurobehavioral outcomes in PWH. Participants for these analyses were assessed between 2010 and 2024. Participants with severe medical (e.g., traumatic brain injury with permanent neurological sequelae) or psychiatric (e.g., active psychosis) conditions that confounded attribution of the cause of neurocognitive impairment to HIV were excluded before cognitive assessment (30). Participants were stratified into four groups according to HIV and diabetes status: HIV+/DM+, HIV+/DM−, HIV−/DM+, and HIV−/DM−, with 50 participants in each group.

The UCSD Human Research Protections Program approved this study. All participants provided written informed consent for using their data in research.

### Neurobehavioral assessments

All participants underwent a neurocognitive assessment that included a comprehensive battery of tests targeting seven neurocognitive domains commonly impacted by HIV (31). The assessed domains were executive function, processing speed, learning, delayed recall, working memory, verbal fluency, and motor function (30). Raw test scores were transformed into T-scores adjusted for age, education, gender, race, and ethnicity based on normative samples of PWoH, with a mean of 50 and a standard deviation of 10 (32). Lower T-scores reflect poorer performance, and scores <40 (≥1 SD below the mean) suggest clinically meaningful impairment. T-scores across tests within a domain were averaged to generate domain-specific T-scores and across all tests to create a global T-score.

### Laboratory assessment

HIV infection was diagnosed by a commercial diagnostic test performed at a Clinical Laboratory Improvement Amendments (CLIA) certified lab. Additional lab assessment included a comprehensive metabolic panel, complete blood counts, rapid plasma reagin tests, CD4+ T cell counts, and plasma HIV RNA, which was quantified by reverse transcriptase-polymerase chain reaction (Roche Amplicor, version 1.5). AIDS diagnosis was established based on accessible clinical and immunological data, specifically defined as having a nadir CD4+ T cell count of less than 200 cells/μL or a history of a clinical condition meeting the criteria for AIDS (33). Participants in the diabetes group either self-reported diabetes mellitus or were taking an antihyperglycemic medication. Glycosylated hemoglobin (HbA1c) was measured in EDTA-anticoagulated whole blood at the UC San Diego Health Center for Advanced Laboratory Medicine Chemistry Laboratory using an immunoturbidimetric assay.

The GlycA 1H-nuclear magnetic resonance (NMR) signal is derived from the N-acetyl methyl protons of N-acetylated carbohydrate side chains of serum glycoproteins (predominantly, α1-acid glycoprotein, haptoglobin, α1-antitrypsin, α1-antichymotrypsin, and transferrin) (Labcorp, Morrisville, NC) (34). BCAAs and gut-related metabolites were measured by NMR spectroscopy using a Vantera^®^ NMR Clinical Analyzer (Labcorp, Morrisville, NC) (17, 35, 36). The DRI score was developed to enhance the predictive performance of LP-IR for type 2 diabetes by incorporating BCAA levels, which have been associated with diabetes risk through potentially new pathways. The regression coefficients derived from this model served as weighting factors in the following equation: DRI = 0.0167 (LP-IR) + 1.907 [ln (valine + 2 × leucine)]. For clinical application, DRI values were scaled to a 1–100 range, using the 1st and 99th percentile values to define the lower and upper bounds (9, 11). Metabolic Vulnerability Index scores were calculated from Inflammation Vulnerability Index and Metabolic Malnutrition Index scores as previously described (37). The MVX is a test that produces a multimarker score (1-100) that assesses a patient’s relative risk of death in 3–5 years, independent of age and other clinically relevant risk factors. Higher MVX scores indicate higher mortality risk. All these tests are clinically available.

### Statistical analysis

Statistical analyses were performed using JMP Pro (version 18, SAS, Cary, NC). For skewed distributions, values were transformed to reduce skewness (e.g., log_10_ or square root transformation). Demographic and clinical characteristics were summarized and presented as count (percentage), mean (standard deviation), or median (interquartile range) across the four subgroups. Categorical data were compared through the Chi-square test. Group comparisons were performed using the independent sample t-test (for normally distributed data) or the Mann–Whitney test (for non-normally distributed data). The primary outcome was global T-score, and the primary hypothesis-driven exposures were DRI, LP-IR, GlycA, and betaine. Analyses involving BCAAs, the Metabolic Vulnerability Index, individual cognitive domains, recursive partitioning, or subgroup interactions were considered secondary. The relationship of global mean T-scores with DRI, LP-IR, and betaine was assessed using linear regression. For figures displaying fitted regression lines, the values shown next to each line represent the F-statistic, degrees of freedom, and corresponding P-value from the unadjusted linear regression model within the indicated subgroup. The F-statistic tests whether the fitted regression slope differs significantly from zero; the values in parentheses indicate the numerator and residual degrees of freedom, respectively. These figure-level statistics are provided to summarize the unadjusted subgroup-specific associations shown graphically, whereas adjusted regression estimates are reported in the Results where applicable. Although the global T-scores were demographically corrected for age, education, sex, race, and ethnicity during score derivation, age, sex, AIDS status, CD4 Nadir, plasma HIV RNA, ART regimens (NRTI/II, NNRTI/II, 4+ class, 3-class, etc), and body mass index (BMI) were included in multiple regression models as potential confounding variables and remained in the models if their p-values were less than 0.15 in the backward elimination process. Interaction was then included in the models to examine the influence of HIV on the relationship between neurocognitive performance and diabetes-related variables and gut microbiomes. When an interaction term was statistically significant, HIV-stratified regression estimates were reported. Interaction analyses evaluated effect modification and did not constitute mediation analyses. To characterize the sensitivity of the interaction analyses given the available sample, we computed the minimum interaction effect detectable at 80% power. For each adjusted interaction model, we derived the smallest HIV-by-marker interaction, expressed as Cohen’s f² and the corresponding partial correlation, detectable at a two-sided α of 0.05 with 80% power, based on the standard error and residual degrees of freedom of the fitted model. To further evaluate the stability of the significant interaction terms, we performed bootstrap resampling. For each interaction model, we drew 5000 bootstrap samples by resampling participants with replacement, refitted the model in each sample, and constructed 95% bias-corrected bootstrap confidence intervals for the HIV-by-marker interaction coefficient.

All statistical tests were two-sided, and P < 0.05 was considered statistically significant. P-values in pairwise analyses were adjusted using Tukey’s honestly significant difference test to account for family-wise type I error multiple comparisons. As a secondary, exploratory analysis, we used recursive partitioning (decision-tree analysis) to identify data-driven thresholds in continuous insulin resistance markers that best discriminated global cognitive performance. To identify complex, non-linear relationships and interactions among predictors, we utilized recursive partitioning. This non-parametric approach recursively splits the predictor space into mutually exclusive, increasingly homogeneous subgroups, maximizing the variance explained in the outcome.

## Results

### Participant characteristics

(**Table 1**): The study included 200 participants divided into four groups of 50 participants each: people with HIV and diabetes mellitus (HIV+/DM+), people with HIV without diabetes mellitus (HIV+/DM−), people without HIV with diabetes mellitus (HIV−/DM+), and people without HIV without diabetes mellitus (HIV−/DM−). Participants were between 19 and 92 years of age (mean = 56) and predominantly men (71%). About half of PWH were non-Hispanic White (45%), 15.5% were Black, and 38% were Hispanic. The median nadir CD4+ T-cell count was 200/µL, and the median current CD4 was 623 cells/μL; 51% had an AIDS diagnosis. 87% of PWH in this study were currently on ART, and 77% were virally suppressed (plasma HIV RNA ≤ 200 copies/mL). Among PWH, those with diabetes had lower current and nadir CD4 counts. As expected, diabetes was associated with higher HbA1c, DRI, LP-IR, BCAAs, and GlycA. Notably, HbA1c and GlycA were lower in HIV+/DM+ than in HIV–/DM+, possibly reflecting closer medical monitoring and treatment engagement in PWH. Global T-scores trended lower in diabetics.

**Table 1:** Demographic and clinical summary by HIV and diabetes mellitus (DM) group.

Demographic and clinical variables	HIV+/DM+ N= 50	HIV+/DM- N= 50	HIV-/DM+ N= 50	HIV-/DM- N= 50	P-value
Age, mean±SD	58.6±10	52.9±14	57.8±9	54.5±15	NS
Male, N (%)	37 (74)	38 (76)	35 (70)	33 (66)	NS
Ethnicity/race, N (%)					NS
Black	9 (18)	8 (16)	6 (12)	8 (16)	
Hispanic	16 (32)	19 (38)	24 (48)	17 (34)	
White	25 (50)	23 (46)	18 (36)	25 (50)	
BMI, mean±SD	27.4±8.4	25.8±6.6	28±8.5	28±5.5	NS
Education, mean±SD	13±3.4	13.5±3.2	13±3.5	14±2.9	NS
Current CD4, median (IQR)	582 (474-817)	650 (502-990)	–	–	0.01
CD4 nadir, median (IQR)	148 (47.5-309)	226 (91.5-462)	–	–	0.04
Plasma viral load ≤ 200 copies/mL, N (%)	39 (78)	38 (76)	–	–	NS
Antihyperglycemic medication count, mean±SD	0.88±0.09	–	1.18±0.09	–	< 0.001
Global T-score, mean±SD	47.9±7.6	48.6±7.08	46±6.1	49.7±6.3	0.052
DRI, mean±SD	57.7±20.8	40.2±20.1	61.3±22.48	44.7±19.4	< 0.001
LP-IR, mean±SD	64.22±18.1	55±21.5	58.3±21.1	52±21.1	0.02
HbA1_C_ (mmol/mol), mean±SD	6.62±1.6	5.52±0.4	7.8±1.8	5.6±0.5	< 0.001
GlycA, mean±SD	444.4±85.2	408.3±77.7	452.3±79.5	416.1±65.2	0.01
Betaine, mean±SD	39±13.2	41.6±11.7	40±12.3	45.5±15	NS
Valine, mean±SD	246.5±69.3	204.6±49	273.2±79	225.3±47.2	< 0.001
Leucine, mean±SD	149.3±60.4	112.2±39	172.2±64.2	131.3±35.3	< 0.001
Isoleucine, mean±SD	64.6±23	56.2±24.2	80±38.1	58.7±18.7	< 0.001
Inflammatory Vulnerability Index, mean±SD	46.2±13.7	42.6±14	45.5±11.5	41±10	NS
Metabolic Vulnerability Index, mean±SD	47.4±12	46.6±11.7	48±9	44.1±8	NS
Metabolic Malnutrition Index, mean±SD	50.3±7.3	53.3±7.3	51.8±7.7	50.7±8.4	NS

NS, Not significant; IQR, Interquartile Range; BMI, body mass index; DRI, Diabetes Risk Index; LP-IR, Lipoprotein Insulin Resistance Index.

### Effect of metabolic dysfunction on cognitive function

**Figure 1:**
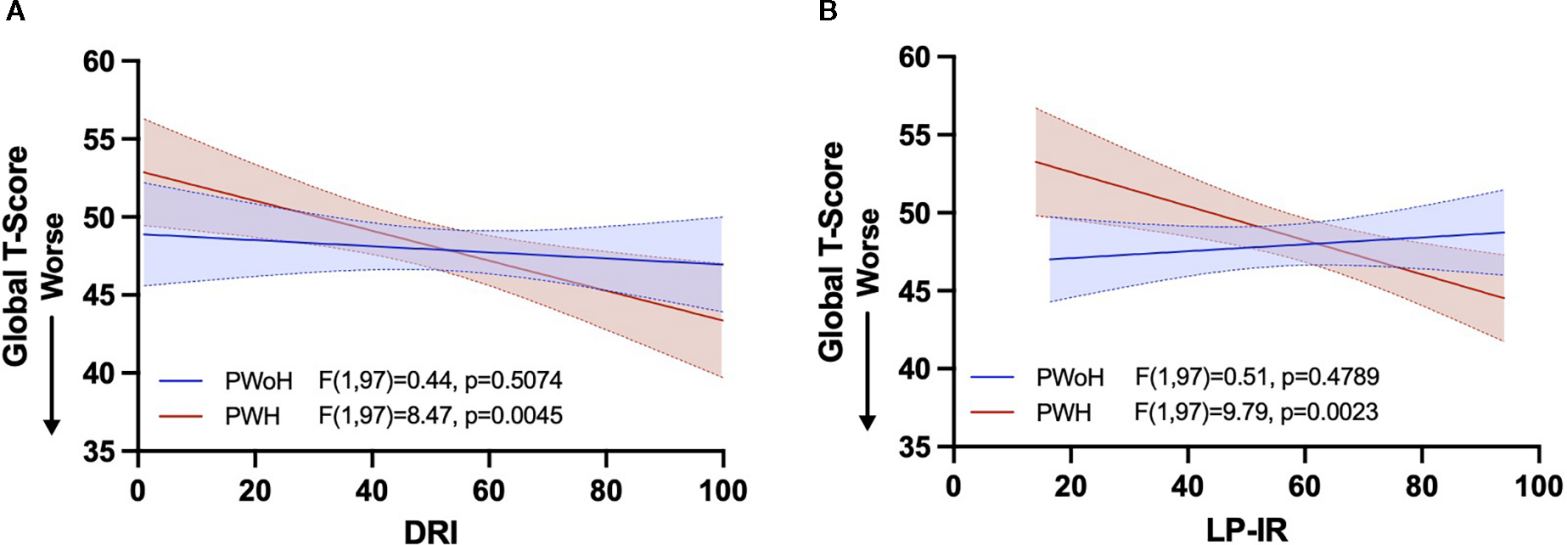
HIV modified the relationship between the global T-score and DRI and LP-IR. The values shown next to each fitted line represent the F-statistic, degrees of freedom, and corresponding P-value from the unadjusted linear regression model within each HIV-status group. **(a)** The relationship between global T-score and DRI by HIV status. **(b)** Relationship between global T-score and LP-IR, stratified by HIV status (interaction P=0.007). Lines indicate fitted regression relationships for PWH (blue) and PWoH (red). Lower T-scores reflect poorer cognitive performance.

Linear regression analyses revealed a significant negative association between the DRI and global T-score, indicating that greater metabolic dysfunction is associated with worse cognitive performance in PWH. [**Figure 1a**, standardized (std) β (95% confidence interval) = -0.28 (-.48, -0.09), P = 0.004]. In contrast, no significant association was found in PWoH [std β (95% confidence interval) = -0.06 (-0.15, 0.27), P = 0.56]. The significant association remained in PWH after adjusting for age, sex, BMI, CD4 nadir, diabetes status, AIDS status, and plasma viral load. [std β (95% confidence interval) = -0.32 (-0.53, -0.11), P = 0.003] Similarly, higher LP-IR scores were significantly associated with lower global T-scores in PWH after adjusting for covariates in multivariable analysis [**Figure 1b**, std β (95% confidence interval) = -0.46 (-0.68, -0.23), P < 0.001]. In contrast, no significant relationship was observed in PWoH (std β [95% confidence interval] = 0.16 [-0.06, 0.39], P = 0.15). In multivariable models, a significant interaction between LP-IR and HIV status on global T-scores was identified (P=0.007), but an interaction between DRI and HIV status was not significant (P=0.08). Regression models showed that higher HbA1c was associated with lower global T-scores among PWH (std β [95% confidence interval] = –0.28 [-0.53, -0.04], P = 0.02), independent of age, sex, nadir CD4, plasma viral loads, BMI, and diabetes status. Given the significant interaction between HIV status and LP-IR in relation to global T-score, we further explored potential clinically meaningful thresholds using recursive partitioning analysis. This approach identified an LP-IR value of 78 as the optimal cut point for discriminating cognitive performance among PWH. Among PWH, individuals with LP-IR ≥ 78 (n = 21) demonstrated significantly lower global T-scores compared to those with LP-IR < 78 (n = 78). The magnitude of this difference corresponded to a large effect size (Cohen’s d = 0.90), indicating substantial cognitive vulnerability associated with elevated insulin resistance in PWH. We performed similar threshold analyses for the DRI, stratified by HIV status. Recursive partitioning identified different optimal cut points by group: a DRI value of 37 for PWH and 68 for PWoH. Among PWH, individuals with DRI ≥ 37 exhibited significantly lower global T-scores compared to those below this threshold (Cohen’s d = 0.65, P = 0.002), representing a moderate-to-large effect size. In contrast, among PWoH, individuals with DRI ≥ 68 showed lower global T-scores compared to those below this value (Cohen’s d = 0.44, P = 0.053). Because this threshold was identified and evaluated in the same cohort without cross-validation, it should not be interpreted as an established clinical cutoff.

### Effect of gut-related metabolite markers on metabolic function

**Figure 2:**
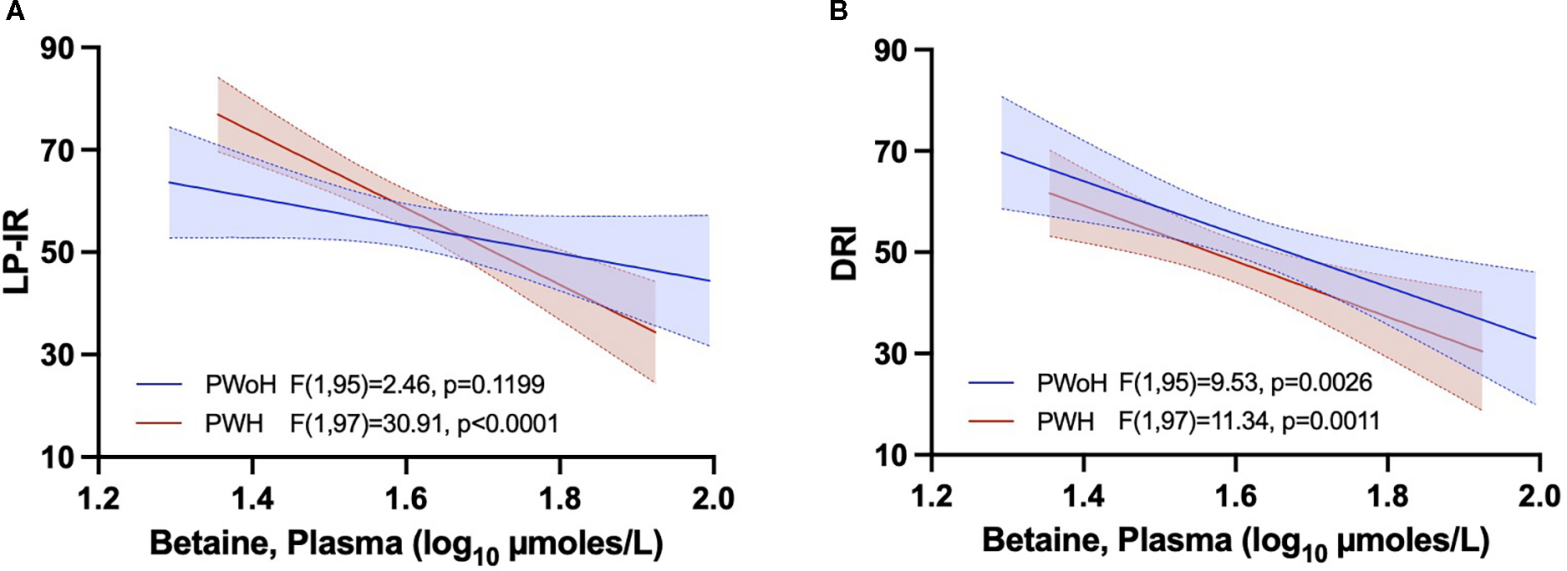
**(a)** The inverse correlation between plasma Betaine and LP-IR stratified by HIV status; **(b)** the inverse correlation between plasma Betaine and DRI by HIV status. The values shown next to each fitted line represent the F-statistic, degrees of freedom, and corresponding P-value from the unadjusted linear regression model within each HIV-status group.

Higher betaine was significantly associated with lower LP-IR in PWH (**Figure 2a**, std β [95% confidence interval] = -0.47 [-0.66, -0.30], P < 0.001). This association remained significant after adjusting for BMI, sex, diabetes status, plasma viral loads, ART regimen, AIDS status, and CD4 nadir (std β [95% confidence interval] = -0.41 [-0.60, -0.22], P < 0.001). The interaction between HIV status and LP-IR was significant (std β [95% confidence interval] = 0.46 [0.06, 0.86], P = 0.02). Higher betaine was significantly associated with lower DRI in both PWH and PWoH. (P <0.05) These associations remained significant after adjusting for age, sex, BMI, ART regimen, CD4 nadir, plasma viral loads, and diabetes status.

### Effect of gut-related metabolite markers on cognition

**Figure 3:**
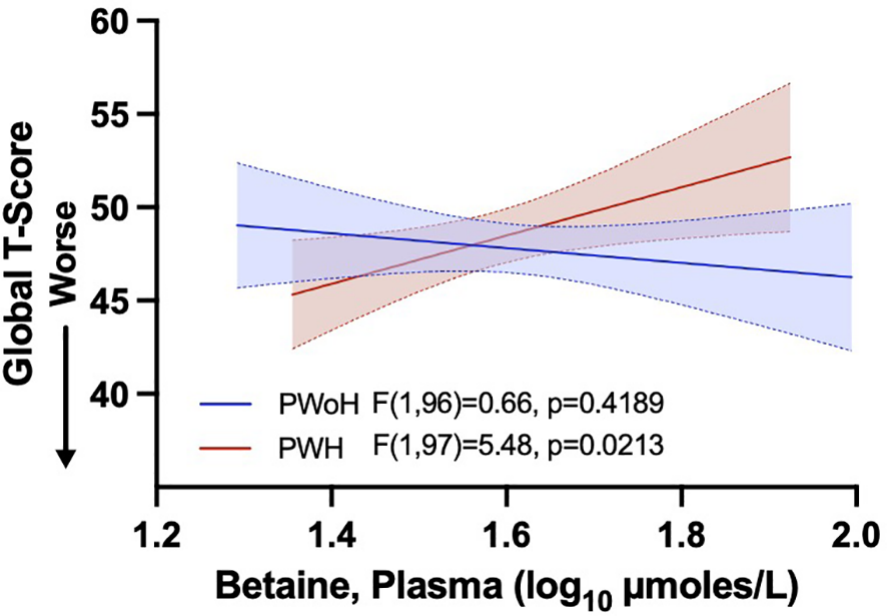
The relationship between the global T-score and betaine by HIV status. The values shown next to each fitted line represent the F-statistic, degrees of freedom, and corresponding P-value from the unadjusted linear regression model within each HIV-status group.

The interaction of HIV status and betaine on global T-scores was significant. (**Figure 3**, std β [95% confidence interval] = -0.21 [-0.39, -0.03], P = 0.01). In PWH, higher betaine was associated with better cognitive performance (std β [95% confidence interval] = 0.26 [0.05, 0.47], P = 0.02). The model was adjusted for age, CD4 nadir, plasma viral loads, diabetes status, AIDS status, and sex, suggesting a potential neuroprotective role.

In a minimum detectable effect analysis, the study had 80% power (two-sided α = 0.05) to detect an HIV-by-marker interaction of approximately Cohen’s f² = 0.04 (partial correlation r ≈ 0.20). The significant HIV-by-LPIR and HIV-by-betaine interactions corresponded to partial correlations of approximately 0.20 and 0.17, respectively, at and near this threshold. Nonparametric bootstrap resampling (5000 resamples) produced interaction estimates consistent with the primary models. The 95% bootstrap confidence intervals were (−0.64 to −0.12) for the HIV-by-LPIR interaction, and (0.02 to 0.62) for the HIV-by-betaine interaction, agreeing closely with the parametric estimates and indicating that both interactions were stable under resampling. These results indicate that the interactions, although detected at or near the limit of the study’s statistical resolution, were robust to resampling, and they are interpreted as exploratory.

Additionally, we determined that people who had hypertension or hyperlipidemia were more likely to have lower plasma betaine levels (std β [95% confidence interval] = -0.89 [-1.42, -0.36], P = 0.001; std β [95% confidence interval] = -0.73 [-1.21, -0.25], P = 0.003, respectively) in PWH, using multivariable logistic regression models, adjusting for age, sex, BMI, plasma viral load, and nadir CD4 count.

### GlycA differences by diabetes status

**Figure 4:**
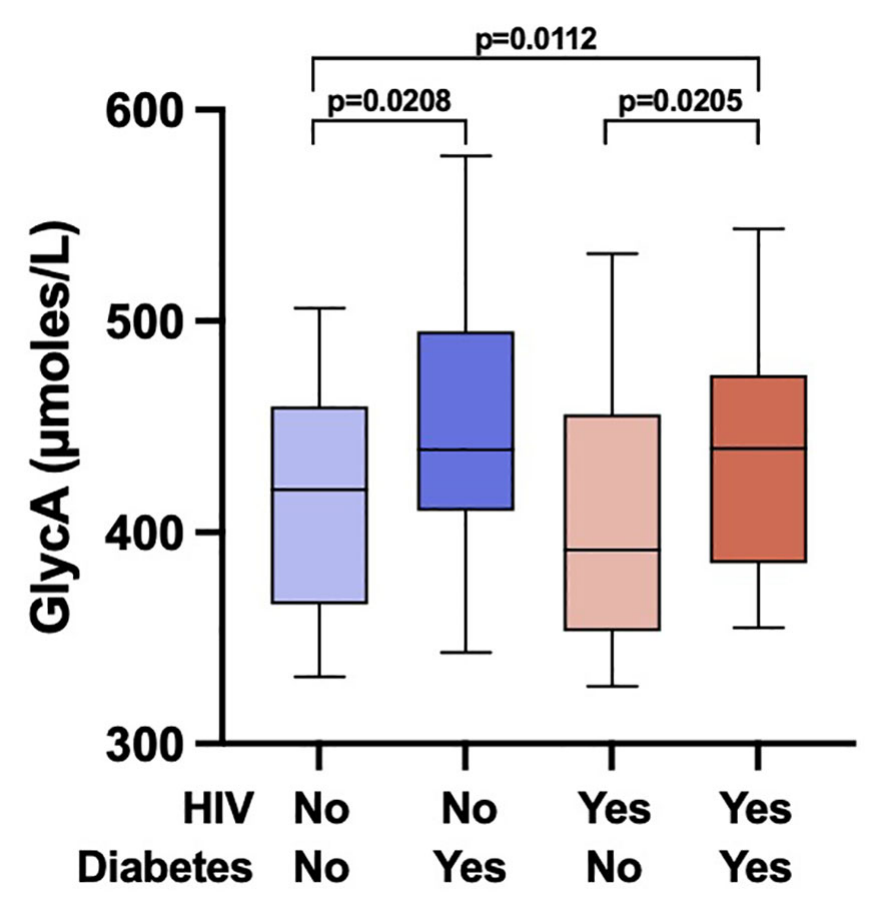
Boxplot of GlycA levels by HIV and diabetes status.

**Figure 4** shows that GlycA levels were significantly higher in individuals with diabetes regardless of their HIV status.

Across HIV and diabetes subgroups, GlycA was consistently associated with markers of metabolic dysregulation. We found that higher GlycA levels were significantly associated with increased Metabolic Vulnerability Index (MVX) scores in all groups (P-values<0.05), higher HbA1_c_ in PWoH, and worse glycemic control in multivariable models, adjusting for age, sex, and BMI (std β [95% confidence interval] = 0.28 [0.10–0.67], P = 0.01). As a strong determinant of HbA1_c_ (p < 0.001), diabetes status affected levels of Leucine, Valine, and Isoleucine, in which their levels were elevated in the diabetic group, compared to non-diabetics (ps < 0.001).

## Discussion

We observed that higher GlycA levels, a marker of systemic inflammation, were significantly elevated in individuals with diabetes, regardless of HIV status. These findings support the role of chronic inflammation as a shared pathophysiological mechanism linking diabetes and cognitive impairment (38). Importantly, GlycA was associated with metabolic vulnerability across HIV and diabetes subgroups, reinforcing its utility as a biomarker of metabolic risk. Our data revealed that higher DRI and LP-IR scores were associated with poorer global cognitive performance in PWH. The significant LP-IR-by-HIV interaction suggests that HIV status may modify the strength of the association between insulin resistance and cognitive performance. However, these findings should not be interpreted as demonstrating that insulin resistance is unrelated to cognition in PWoH.

Impaired insulin signaling may reduce neuronal glucose utilization, disrupt synaptic plasticity, alter neurotransmitter regulation, and increase mitochondrial dysfunction and oxidative stress (39). Peripheral inflammation may be an important contributor to the development and progression of insulin resistance and type 2 diabetes mellitus. Chronic low-grade systemic inflammation can disrupt insulin signaling in insulin-sensitive tissues, including skeletal muscle, adipose tissue, and the liver, thereby promoting insulin resistance and worsening glycemic regulation (40). This HIV-specific vulnerability suggests that chronic immune activation and residual inflammation may amplify the adverse cognitive effects of metabolic dysfunction. Notably, this study is among the first to apply LP-IR and DRI to characterize metabolic–cognitive interactions in HIV, underscoring the novelty and translational potential of these NMR-based markers for identifying early metabolic risk in this population. Our findings align with growing evidence that PWH, even when well-controlled on ART, are uniquely susceptible to insulin resistance-related cognitive decline (41, 42). Additionally, the interaction analysis suggests that HIV status may modify the relationship between metabolic dysfunction and cognition, indicating a potentially greater vulnerability in this population. These findings highlight the broader impact of metabolic health on brain function and suggest that metabolic dysfunction may be an important target for interventions aimed at preserving cognitive health, especially in populations with chronic conditions like HIV.

This study found that among gut-related metabolites, betaine emerged as a potentially anti-inflammatory and protective factor. Higher plasma betaine levels were consistently associated with lower DRI and LP-IR scores, with stronger effects in PWH. Furthermore, betaine was positively associated with global cognitive performance in PWH, suggesting its dual role in improving metabolic health and preserving cognition (43). Other studies suggest potential cognitive benefits of betaine. For instance, betaine supplementation has been shown to prevent cognitive dysfunction by suppressing hippocampal microglial activation in chronically socially isolated mice. Additionally, betaine has demonstrated neuroprotective properties in animal models, such as alleviating oxidative stress and cognitive impairments induced by amyloid-beta peptides (44, 45).

BCAAs were also associated with metabolic indices, though their cognitive correlates were less robust (46). Elevated BCAA levels are known to impair insulin signaling and may contribute to systemic inflammation (46). Further investigation is warranted to understand whether BCAA dysregulation contributes to neurocognitive decline, particularly in PWH. The relationship between BCAAs and cognitive function appears to be complex. Some studies suggest that higher circulating BCAA levels are associated with an increased risk of Alzheimer’s disease (AD). For instance, research has demonstrated that individuals with AD have elevated circulating BCAAs compared to healthy controls, and dietary restriction of BCAAs in animal models delayed cognitive decline and reduced AD-related pathology. Conversely, other studies have found decreased circulating BCAA levels in individuals who developed AD, indicating a potential protective role (47, 48). Furthermore, studies have shown that BCAA supplementation can influence cognitive performance. For example, a study in healthy volunteers found that BCAA intake affected neuroendocrine responses and spatial recognition memory, suggesting an impact on cognitive processes (48, 49).

This cross-sectional study cannot establish causality, and the findings should be interpreted within this context. Moreover, there is a potential for reverse causality and bi-directionality, where metabolic dysfunction may both contribute to and result from cognitive decline, creating a feedback loop that reinforces neurocognitive vulnerability over time. Self-reported diabetes diagnosis may have introduced misclassification bias, though biomarker-based assessments partially mitigate this concern. The predominance of men may limit generalizability to women and preclude adequately powered sex-specific analyses. Additionally, microbiome composition via fecal microbiome sequencing was not directly assessed, limiting our ability to link specific microbial taxa with circulating metabolites and cognitive outcomes. Other limitations include a lack of information on potential lifestyle confounders such as diet quality, physical activity, and sleep, and possible imbalance in education or socioeconomic status, which could influence both metabolic health and cognitive outcomes. Detailed information regarding medication adherence and longitudinal glycemic control was not consistently available. These factors may have contributed to differences in HbA1c and GlycA between the diabetic HIV groups and limited our ability to explain those differences. Because the study was powered to detect main effects rather than effect modification, the interaction analyses were exploratory. The significant interactions were detected at or near the lower limit of the study’s statistical resolution. Bootstrap confidence intervals for these interactions were consistent with the primary estimates, but their magnitudes may be imprecise or overestimated and require confirmation in larger, independent cohorts.

Future work should investigate these relationships over time to determine causality. Longitudinal analyses will help clarify whether insulin resistance and inflammation precede neurocognitive decline in PWH or result from shared underlying mechanisms. Future studies should incorporate validated dietary assessments, including measures of betaine-, choline-, and protein-rich food intake, to determine whether circulating betaine and BCAAs independently relate to cognition or instead serve partly as markers of healthier dietary and lifestyle patterns. The sample-derived LP-IR threshold of 78 is above the clinical threshold of 68 commonly used to indicate elevated insulin resistance and increased diabetes risk. This suggests that the cognitive difference observed in our cohort occurred within a range reflecting relatively high metabolic risk. However, the established threshold of 68 was developed for cardiometabolic and diabetes-risk assessment, not cognitive outcomes. Moreover, because the cutoff of 78 was derived without internal or external validation, it may be sensitive to the characteristics of this sample. It should therefore be viewed as an exploratory finding requiring cross-validation and replication in independent cohorts before being considered for cognitive risk stratification.

Experimental studies are needed to unravel the molecular pathways by which metabolic markers, such as LP-IR, DRI, GlycA, and gut-related metabolites like betaine, impact brain function. Integrating neuroimaging, cerebrospinal fluid biomarkers, and peripheral blood analyses may provide deeper mechanistic insights. Given the observed associations between metabolic dysfunction and cognition, future trials should assess whether improving metabolic health through dietary, pharmacologic, or lifestyle interventions can mitigate cognitive impairment in PWH. From a therapeutic perspective, betaine represents a promising and feasible intervention target. It can be administered safely as a dietary supplement or through increased dietary intake of betaine-rich foods (e.g., whole grains, spinach, and beets), and has shown good tolerability in metabolic and cardiovascular studies. Probiotic or microbiome-directed strategies aimed at enhancing endogenous betaine production may also hold potential to improve both metabolic and cognitive outcomes in PWH. Betaine supplementation, for example, holds promise as a dual modulator of insulin sensitivity and neuroprotection.

## Conclusion

This study underscores the complex interplay between metabolic dysfunction, systemic inflammation, and cognitive performance, particularly among PWH. Our findings highlight GlycA as a robust marker of inflammation linked to diabetes and metabolic vulnerability. DRI and LP-IR scores were specifically associated with cognitive impairment in PWH. These results suggest that chronic immune activation in HIV may exacerbate the cognitive consequences of metabolic disturbances. Additionally, the protective associations observed with betaine in both people with and without HIV suggest a potential therapeutic avenue for enhancing both metabolic and cognitive health. These findings should not be taken as evidence that metabolic risk factors are unrelated to cognition in PWoH and require confirmation in larger longitudinal cohorts. Although the associations observed cannot establish causality, they lay the groundwork for future longitudinal and mechanistic studies.

## Data Availability

The raw data supporting the conclusions of this article will be made available by the authors, without undue reservation.
